# Development of a Sensory Evaluation Method for Polyphenols via Analysis of Chemical Structure and Organoleptic Properties: A Pilot Study

**DOI:** 10.3390/foods15081409

**Published:** 2026-04-17

**Authors:** Hitomi Nakamura, Moeka Ogata, Takafumi Shimizu, Yasuyuki Fujii, Kenta Aso, Chika Tagata, Vittorio Calabrese, Naomi Osakabe

**Affiliations:** 1 Functional Control Systems, Graduate School of Engineering and Science, Shibaura Institute of Technology, Saitama 337-8570, Japan; bn20020@shibaura-it.ac.jp (H.N.); bn21043@shibaura-it.ac.jp (M.O.); bn20003@shibaura-it.ac.jp (T.S.); 2 SIT Research Laboratories, Shibaura Institute of Technology, Saitama 337-8570, Japan; fujii.yasuyuki.x1@shibaura-it.ac.jp; 3 Central Research Institute, ITO EN, Ltd., Shizuoka 421-0516, Japan; k-aso@itoen.co.jp (K.A.); chika-yokoyama@itoen.co.jp (C.T.); 4 Department of Biomedical and Biotechnological Sciences, University of Catania, 95124 Catania, Italy; vittorio.calabrese@unict.it

**Keywords:** polyphenol, sensory analysis, acidity, bitterness, astringency

## Abstract

Polyphenols are plant metabolites with potent physiological activities. Despite their known bitterness and astringency, their specific sensory characteristics remain poorly understood. To clarify the relationship between polyphenol chemical structures and sensory profiles, we developed a sensory evaluation protocol for a young-adult panel. Following four days of intensive monthly training, the panel achieved proficiency in distinguishing bitterness, astringency, and acidity. Four polyphenols with distinct structures—gallic acid, quercetin hydrate, epigallocatechin gallate (EGCG), and a procyanidin-rich fraction (PRF)—were evaluated using flavor profile analysis (FPA) and 3-Alternative Forced Choice (3-AFC) tests for their qualitative properties, and quantitative descriptive analysis (QDA) for their quantitative properties. The results showed that gallic acid was acidic, and EGCG was bitter and astringent, with the intensity being concentration-dependent. In contrast, quercetin hydrate did not show any significant sensory properties. This methodology facilitates the elucidation of the relationship between polyphenol structures, and their organoleptic properties and subsequent findings help to further clarify the role of polyphenol–taste receptor interactions in health benefits.

## 1. Introduction

Polyphenols are a diverse group of plant secondary metabolites with more than 8000 species identified to date, ranging in molecular weight from 110 to 30,000 and varying in physicochemical properties [1]. Polyphenols are non-volatile compounds and have been shown to have a bitter taste, and some compounds have an astringent taste [2,3]; however, their precise sensory characteristics and the mechanisms by which they are expressed remain unclear.

A negative correlation has been demonstrated between the consumption of polyphenol-rich foods and cardiovascular disease [4], neurodegenerative diseases [5,6], and age-related deterioration of sensory organs [7,8], as evidenced by epidemiological studies. It has been hypothesized that individuals who habitually consume astringent and bitter foods may exhibit a reduced risk of type 2 diabetes and cardiovascular disease [9,10].

Bitterness is known to be recognized by taste receptor 2 (T2R) expressed in type III taste cells [11,12,13]. These nerve impulses are transmitted via the facial, glossopharyngeal, and vagus nerves. They are then relayed to the nucleus of the solitary tract (NST) in the brainstem and sent to the insular cortex and medial frontal lobe via the thalamus, where bitterness is recognized [14,15]. Recent reports have indicated the expression of taste receptors, including T2R, not only in the oral cavity but also in the secretory cells of the digestive tract [3]. It has long been established that taste receptors expressed on gastrointestinal secretory cells are activated by sweet, umami, and bitter taste substances, and secrete gastrointestinal hormones such as incretin and cholecystokinin to maintain glucose tolerance and to control gastrointestinal motility [16,17]. Numerous intervention studies have documented that the ingestion of polyphenols, either as a single dose or as repeated doses, has been observed to attenuate the increase in blood glucose levels subsequent to an oral glucose tolerance test [3,18]. Furthermore, an increase in the blood concentration of glucagon-like peptide (GLP)-1, a type of incretin, has been observed, suggesting that the bitter taste of polyphenols may be involved in this change.

Astringency is also a stimulus caused by specific polyphenols, and its recognition mechanism is unknown. It has been thought that polyphenols bind to salivary proteins to form particles, which then scrape the oral mucosa, and that this stimulus is transmitted to the central nervous system via mechanoreceptors [19]. Conversely, recent studies have reported that the astringent stimuli of polyphenols excite the central nervous system even in the absence of salivary proteins. It has also been suggested that transient receptor potential (TRP) channels expressed in the oral cavity and digestive tract may recognize astringency [20,21,22]. Specifically, procyanidins, which are catechin oligomers that are pigmented components, are potent astringents. Furthermore, they are characterized by high bioactivity. Specifically, a large-scale, long-term intervention study of procyanidin in elderly subjects reported improvements in hippocampus-dependent cognitive function [23] and a reduced risk of cardiovascular disease [24]. In summary, the bitter and astringent tastes of polyphenols are hypothesized to significantly influence physiological function.

Numerous methods have been established for measuring the sensory characteristics of food components. Qualitatively, flavor profiling analysis (FPA) and 3-Alternative Forced Choice (3-AFC) are widely employed, while quantitatively, quantitative descriptive analysis (QDA) is commonly used [25]. Training panelists is important for measuring the sensory characteristics of food components using these methods and creating quantitative descriptions that can be statistically analyzed. For example, it is known that sensory evaluation of wine, a food rich in polyphenols, is performed by trained sommeliers. Furthermore, the bitterness and astringency of polyphenols are often misidentified, and training is essential for sensory identification, but methods targeting polyphenols are still in the development stage.

In this study, we selected young, healthy male and female panelists and developed a training method to quantitatively evaluate their sensory characteristics. Furthermore, we verified whether the trained panel could identify the typical sensory characteristics of specific polyphenols by combining three methods: FPA, 3-AFC, and QDA.

## 2. Materials and Methods

### 2.1. Materials

Citric acid and gallic acid were purchased from FUJIFILM Wako Pure Chemical Co., Ltd. (Osaka, Japan). Caffeine and quercetin hydrate were purchased from Tokyo Chemical Industry Co., Ltd. (Tokyo, Japan). Potassium aluminum sulfate (PAS) was purchased from Kenkei Pharmaceutical Co., Ltd. (Osaka, Japan). The polyphenols used to evaluate the training outcome of the panelists are listed in the Phenol Explore (http://phenol-explorer.eu/, version 3.6, accessed on 10 April 2025), and compounds with different chemical structures were selected: phenolic acids (gallic acid), flavonoid aglycones (quercetin hydrate), gallic acid-type catechins (epigallocatechin gallate), and condensed tannins (type B procyanidins). Food-grade epigallocatechin gallate derived from green tea leaves (Table 1) or procyanidin-rich fraction (PRF) derived from cocoa (Table 1) were donated from ITO EN Ltd. (Shizuoka, Japan) or Meiji Co., Ltd. (Tokyo, Japan).

The ingredients were added to water to achieve a final concentration of 0.2–0.8 mg/mL. To ensure maximum dispersion, the mixture was sonicated at 37 °C for 10 min and vigorously stirred. While substances other than quercetin hydrate dissolved completely, the solution containing quercetin hydrate was slightly cloudy. To minimize precipitation, the solution prepared at 37 °C was administered to the subjects immediately after preparation.

### 2.2. Ethics

Sensory tests, FPA, 3-AFC, and QDA, were conducted based on previous studies [25,26]. After the test protocol was approved by the Shibaura Institute of Technology Ethics Committee, the sensory test was registered in the Clinical Trial Registration System UMIN (https://www.umin.ac.jp/ctr/new-registration.htm, UMIN000052854, accessed on 15 January 2026). One hour after consuming the specified food or beverage (breakfast and/or lunch), the panelists performed a series of sensory tests. Each series of sensory tests was conducted at least two hours apart. Standard substances for acidity, bitterness, and astringency, or the test compounds, were randomly assigned using an assignment table (generated by computer-generated random numbers). Data was collected anonymously.

### 2.3. Selection of Panelists

**Figure 1:**
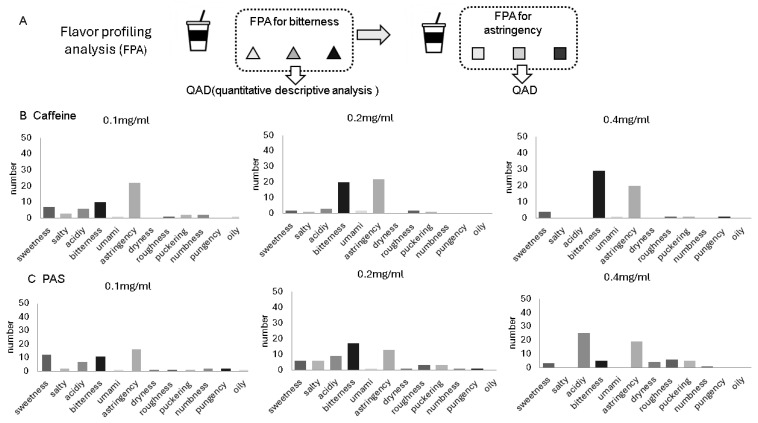
Protocol for panelist selection (**A**) and results of flavor profiling analysis (FPA) and quantitative descriptive analysis (QDA) of three concentrations of caffeine (**B**) or potassium ammonium sulfate (**C**). Results represent the average of potential panelists aged (*n* = 49, 26 males, 23 females, aged 20–28 years).

Before the experiment, written informed consent for voluntary participation and the oral administration of reference solutions and polyphenols was obtained from the candidates (26 males and 23 females, aged 20–28 years). To standardize terminology and measurement scales, a glossary was developed based on the previous literature and utilized for the flavor profile analysis (FPA) test (Table 2). The intensity of each flavor attribute was evaluated using the QDA method (Figure 1) [25,26]. The sensory evaluation was conducted in three integrated phases: screening, training, and quantification. First, the 3-AFC method was utilized to determine individual taste recognition thresholds, ensuring panelist sensitivity. Second, FPA was performed during 21 sessions to establish a consensus on sensory terms and their definitions (Table 2). Finally, QDA was employed to measure the intensity of each attribute for the four polyphenol samples. This sequential integration ensured that the quantitative data (QDA) were based on verified sensory capabilities (3-AFC) and a unified sensory language (FPA).

The screening process for panelist selection was conducted as follows: participants were asked to place their mouths on 10 mL of the solution for 10 s, then spit it out to identify the reference standard. Caffeine and PAS were used to represent bitterness and astringency, respectively, at concentrations of 0.1, 0.2, and 0.4 mg/mL. Participants assessed their sensitivity to various stimuli, including bitterness, astringency, sweetness, saltiness, acidity, umami, dryness, roughness, shrinkage, numbness, spiciness, and oiliness (FPA). Furthermore, participants were tasked with distinguishing between the three concentrations and evaluating the intensity of each sensation using a 100 mm Visual Analog Scale (VAS), ranging from 0 (no sensation) to 100 (strongest imaginable sensation).

### 2.4. Training

The number of panelists was determined through a power analysis based on preliminary experimental results. Based on a priori power analysis using G*power (https://www.psychologie.hhu.de/arbeitsgruppen/allgemeine-psychologie-und-arbeitspsychologie/gpower, accessed on 10 April 2025), the total sample size required to achieve a power of 0.80 was calculated to be 7.

**Figure 2:**
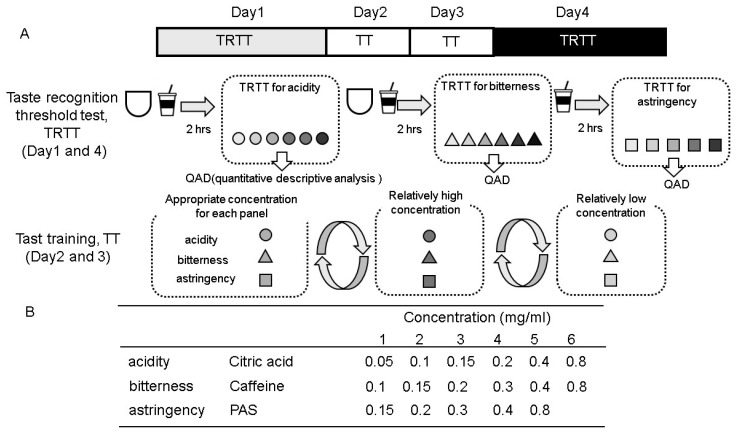
Scheme of the taste training program (**A**) and concentrations of standard taste compounds (**B**) used in this experiment. The training program was conducted over 4 mo, with sessions held for 4 consecutive days for panelists (*n* = 7) each month. Among the standard taste compounds, citric acid and caffeine were used in a 6-step dilution series, while PAS used a 5-step dilution series.

**Figure 3:**
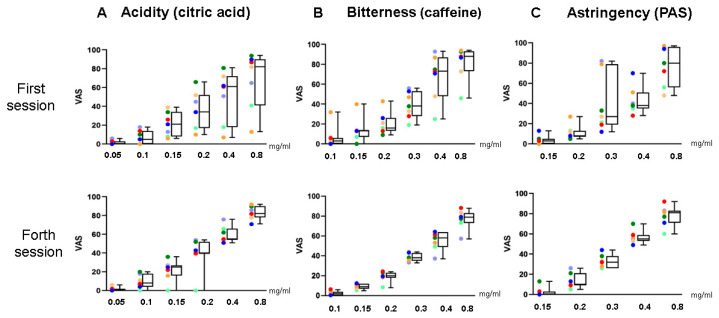
Changes in sensory evaluation of acidity (**A**), bitterness (**B**), and astringency (**C**) during the training period for panelists. The upper row shows data before the first session, and the lower row shows data before the fourth session. Data are presented as box plots (*n* = 7).

As a result, seven subjects (5 males and 2 females, aged 22–25 years) who successfully distinguished both caffeine and PAS at 0.4 mg/mL during a screening test were selected as panelists. The panelists underwent a training program focused on taste identification and the determination of recognition thresholds, following the method described by Otsubo et al. with slight modifications (Figure 2). This program, comprising the taste recognition threshold test (TRTT) and taste training (TT), was conducted over four months, with sessions held on four consecutive days each month. Panel validation was assessed through longitudinal tracking of TRTT and score consistency. By the end of the training (Day 4), the panel demonstrated a marked increase in sensitivity (Figure 3). Furthermore, panel consensus was verified by the convergence of descriptive scores during the 21 training sessions, ensuring that the participants met the proficiency requirements for an analytical sensory panel as defined by ISO guidelines ISO 8589:2007 [27]. The daily protocols were as follows:

− Day 1: The TRTT was conducted using six concentrations of citric acid (0.05, 0.1, 0.15, 0.2, 0.4, and 0.8 mg/mL), six concentrations of caffeine (0.1, 0.15, 0.2, 0.3, 0.4, and 0.8 mg/mL), and five concentrations of PAS (0.15, 0.2, 0.3, 0.4, and 0.8 mg/mL).
− Days 2 and 3: Participants underwent TT, where they were repeatedly exposed to three concentration levels—the recognized threshold, one level above, and one level below—in a randomized order. This process continued until the subjects could accurately and consistently identify the tastes.
− Day 4: Subjects evaluated the three flavors using the QDA method, following the same TRTT protocols as on Day 1.

### 2.5. Sensory Evaluation of Four Representative Polyphenols

**Figure 4:**
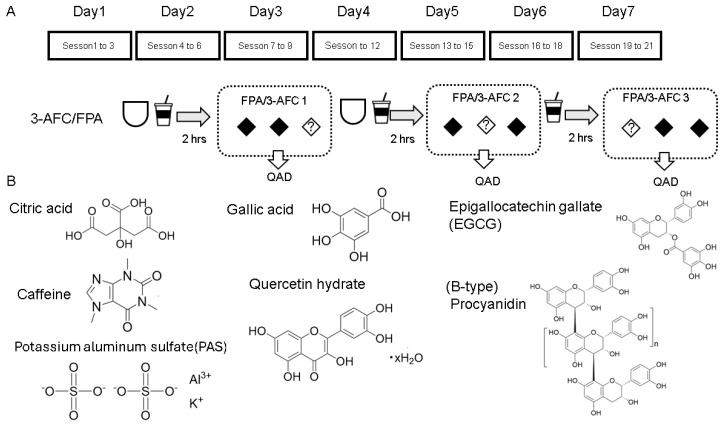
Functional property evaluation method for polyphenols (**A**) and chemical structures of chemicals used (**B**). The panelists evaluated the taste of four types of polyphenols (quercetin hydrate, gallic acid, epigallocatechin gallate, and PRF) with three standard substances at concentrations of 0.2, 0.4, and 0.8 mg/mL.

In addition to the three standard substances previously described, the panelists evaluated four types of polyphenols: quercetin hydrate, gallic acid, epigallocatechin gallate (EGCG), and a PRF (expressed as (−)-epicatechin equivalents). Each was tested at concentrations of 0.2, 0.4, and 0.8 mg/mL. All sensory testing was performed in a dedicated sensory laboratory equipped with individual partitioned booths, in accordance with ISO 8589 standards [27]. The environment was strictly controlled: the room temperature was maintained at 24 ± 1 °C, and the area was kept free from extraneous odors and noise to ensure maximum concentration. To eliminate visual bias and ensure the objectivity of the panelists, standardized white lighting was used throughout the sessions. Furthermore, to prevent interaction and mutual interference, panelists were physically separated by partitions, and samples were presented in a randomized order with three-digit codes. This experimental setup ensured that each assessment was independent and free from psychological or environmental distractions. The evaluations were conducted three times daily—two hours after breakfast, two hours after lunch, and two hours after the second session—over a seven-day period in a randomized order. The trained panelists were instructed to rinse their mouths with 10 mL of three separate solutions (comprising two water blanks and one sample/standard), holding each in the mouth for 10 s before spitting it out. Participants were then tasked with identifying the sample that differed from the others using the 3-AFC and rating the intensity of the sensation using the QDA method (Figure 4).

### 2.6. Data Analysis and Statistical Methods

All statistical analyses were conducted using GraphPad Prism 10 software (GraphPad Software, San Diego, CA, USA). For the sensory evaluation of polyphenols, data were analyzed using the Kruskal–Wallis test, followed by Dunn’s multiple comparison test as a post hoc analysis to determine significant differences between groups. We selected this statistical method because sensory evaluation data, by its very nature, exhibits a non-normal distribution and high variability, and because it is also effective for small sample sizes and ordinal scale analysis. A *p*-value of less than 0.05 (*p* < 0.05) was considered statistically significant.

## 3. Results

### 3.1. Results for the Selection of Panelists

The preliminary test results for panelist selection are presented in Figure 1. When a three-step dilution series of caffeine was used as a bitter reference, bitterness intensity increased in a dose-dependent manner. Although astringency was perceived at all concentrations of PAS, no clear dose-dependency was observed. Notably, participants also reported perceiving bitterness and acidity in the PAS solutions, with the latter demonstrating a dose-dependent effect. These results suggest that untrained subjects were unable to clearly distinguish between different oral sensations or lacked a precise understanding of the descriptive terminology. Based on these findings, we determined that a comprehensive training program focusing on acidity, bitterness, and astringency was essential to ensure the reliability of the sensory evaluation.

### 3.2. Taste Threshold Training

Seven selected panelists underwent a four-month taste training program (one session per month) according to the protocol shown in Figure 2. Figure 3 illustrates the changes in taste thresholds for acidity (citric acid, Figure 3A), bitterness (caffeine, Figure 3B), and astringency (PAS, Figure 3C) recorded during this period. In each figure, the upper graph represents the results from the first session, while the lower graph displays those from the first day of the fourth session.

By the fourth session, a significant training effect was observed, as evidenced by a marked reduction in recognition thresholds and a narrowing of the inter-quartile ranges in the box plots. This improvement suggests that the intensive 21-session training facilitated perceptual learning, which enhanced the panelists’ ability to discriminate subtle gustatory stimuli from background noise. Notably, the stabilization of thresholds for bitterness and astringency—attributes often characterized by higher inter-individual variability—confirmed that the panelists had developed a unified sensory scale. This longitudinal tracking of TRTT and score consistency ensured that the participants met the proficiency requirements for an analytical sensory panel, functioning as a calibrated instrument for the subsequent polyphenol evaluation.

### 3.3. Evaluation of the Sensory Perception of Polyphenols

Four representative polyphenols with chemically distinct structures were selected for the study, and 3-AFC/FPA were conducted as illustrated in Figure 4. Table 3 compares the preliminary observations made before training with QDA results for each compound at 0.4 mg/mL after the fourth training session.

Initially, preliminary observations indicated that panelists demonstrated ambiguity in recognizing sensory terms and discriminating between attributes, particularly in distinguishing between bitterness and astringency, which are often confused by untrained individuals. However, following the fourth training session, attribute discrimination became more evident, and variability in thresholds diminished significantly. This improvement was most pronounced in the perception of bitterness, suggesting that systematic exposure to reference standards successfully calibrated the panelists’ internal scales for complex polyphenolic compounds.

**Figure 5:**
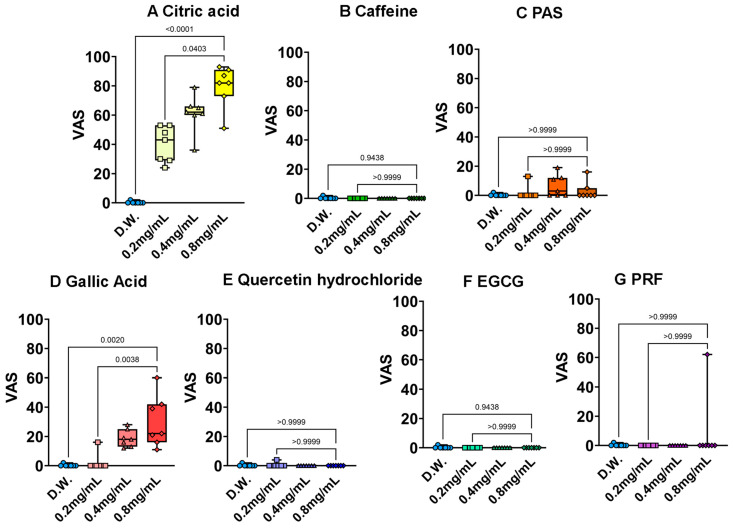
Results of polyphenol acidity measurement by 3-AFC and QDA methods. (**A**): Citric acid, (**B**): caffeine, (**C**): potassium aluminum sulfate (PAS), (**D**): gallic acid, (**E**): quercetin hydrate, (**F**): epigallocatechin gallate (EGCG), (**G**): PRF. Each value shows the mean and standard deviation calculated from VAS results (*n* = 7). Statistical analysis was performed using the Kruskal–Wallis test, and Dunn’s multiple comparison test was used for post hoc comparisons.

Figure 5 presents the results of the acidity evaluation. Gallic acid exhibited potent, dose-dependent acidity, showing a profile remarkably similar to the positive control, citric acid. This phenomenon is chemically justified by its structure as a low-molecular-weight phenolic acid; the free carboxyl group dissociates in aqueous solution, releasing protons ($H^+$) that directly stimulate acid-sensing ion channels (e.g., OTOP1) on the tongue. In contrast, EGCG and the PRF, which lack such acidic functional groups, did not exhibit significant acidity. The absence of acidity in quercetin hydrate is also consistent with its neutral polyphenolic nature and limited molecular interaction due to poor solubility.

**Figure 6:**
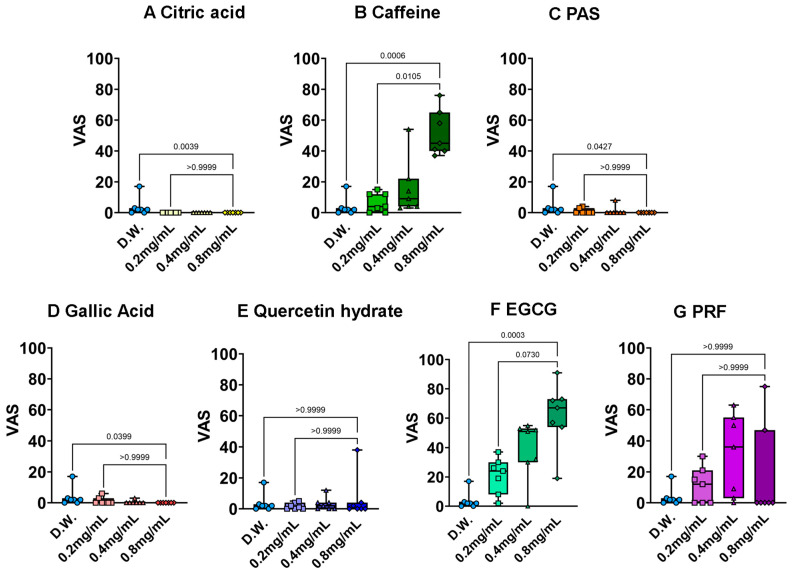
Results of polyphenol bitterness measurement by 3-AFC and QDA methods. (**A**): Citric acid, (**B**): caffeine, (**C**): potassium aluminum sulfate (PAS), (**D**): gallic acid, (**E**): quercetin hydrate, (**F**): epigallocatechin gallate (EGCG), (**G**): PRF. Each value shows the mean and standard deviation calculated from VAS results (*n* = 7). Statistical analysis was performed using the Kruskal–Wallis test, and Dunn’s multiple comparison test was used for post hoc comparisons.

**Figure 7:**
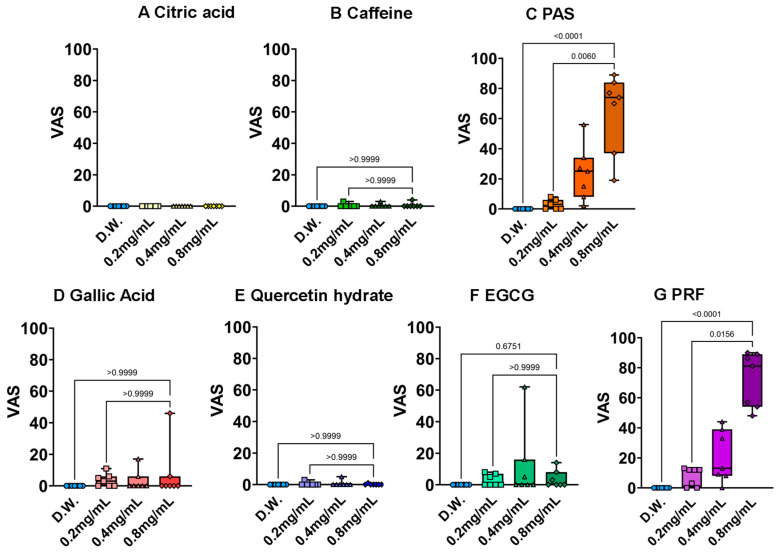
Results of polyphenol astringency measurement by 3-AFC and QDA methods. (**A**): Citric acid, (**B**): caffeine, (**C**): potassium aluminum sulfate (PAS), (**D**): gallic acid, (**E**): quercetin hydrate, (**F**): epigallocatechin gallate (EGCG), (**G**): PRF. Each value shows the mean and standard deviation calculated from VAS results (*n* = 7). Statistical analysis was performed using the Kruskal–Wallis test, and Dunn’s multiple comparison test was used for post hoc comparisons.

The bitterness evaluation results are shown in Figure 6. Both caffeine and EGCG showed clear, dose-dependent bitterness. The bitterness of EGCG is likely mediated by the interaction of its galloyl moiety with specific T2R bitter taste receptors. Interestingly, the PRF was perceived as bitter at all concentrations but lacked clear dose-dependency. This plateau effect might be attributed to the complex composition of the fraction; the presence of various oligomers and monomeric catechins may saturate the bitter receptors even at lower concentrations, or the intense astringency (Figure 7) may have partially masked the perception of bitterness at higher doses. Quercetin hydrate was reported as non-bitter, which we hypothesize is due to its poor aqueous solubility at the tested concentrations, preventing effective molecular interaction with taste receptors in the oral cavity.

The results for astringency are presented in Figure 7. The positive control (PAS) and the PRF are driven by their degree of polymerization; polymerized flavan-3-ols possess a high affinity for salivary proline-rich proteins (PRPs). This interaction leads to the formation of insoluble protein–polyphenol complexes, reducing oral lubrication and triggering the mechanical sensation of “constriction” or “roughness.” While EGCG also showed a slight astringent sensation, the intensity was significantly lower than that of the PRF, emphasizing that higher polymerization or specific oligomeric structures are required to elicit intense astringency.

## 4. Discussion

Although the health-promoting benefits of polyphenols are well established, the potential link between their sensory characteristics and the expression of physiological effects remains poorly understood. To address this gap, the present study aimed to develop a robust methodology for the quantitative assessment of polyphenol sensory profiles, with a specific focus on bitterness and astringency.

As demonstrated in Figure 3, the training protocol developed in this work (Figure 2) proved highly effective, significantly enhancing the panelists’ discriminative capacity and stabilizing their thresholds for acidity, bitterness, and astringency. Leveraging this validated panel, we subsequently evaluated four structurally diverse polyphenols to characterize their distinct sensory properties, fulfilling the primary objective of this research.

For this study, we selected four compounds with distinct chemical structures: a phenolic acid (gallic acid), a flavonoid aglycone (quercetin hydrate), a gallate-type catechin (epigallocatechin gallate, EGCG), and a condensed tannin (B-type procyanidins). Phenolic acids, such as gallic acid, are low-molecular-weight polyphenols characterized by a benzene ring substituted with hydroxyl and carboxylic acid groups [28]. Flavonoids, the most extensively studied of polyphenols, share a common structural characteristic involving two benzene rings (A and B) connected by a three-carbon bridge, commonly referred to as C_6_-C_3_-C_6_ [29]. Flavonoids are categorized into six distinct subclasses—flavones, flavanones, isoflavones, flavonols, chalcones, and anthocyanins—based on modifications to the basic skeleton [30]. Quercetin is a representative flavonol. EGCG is a gallate-type catechin that possesses a specific structure in which a gallate moiety is esterified to a flavanol aglycone. Procyanidins, which are representative condensed tannins, are polymers of catechin units [31].

These polyphenols exhibit significant diversity in both molecular weight and chemical structure and are widely recognized as compounds that elicit bitterness and astringency. However, a comprehensive elucidation of their sensory profiles has yet to be achieved. One primary reason is that sensations such as bitterness and astringency frequently coexist in food matrices, making it difficult for humans to distinctly identify or verbally articulate them during consumption. Consequently, alternative approaches to sensory evaluation, specifically the development of predictive models, have emerged. These models utilize various instrumental techniques, including spectroscopy, fluorescence, capillary electrophoresis, enzymatic assays, microcalorimetry, and turbidimetry, to estimate sensory attributes [32]. Nevertheless, sensory analysis remains a useful tool for evaluating sensory attributes and is currently unparalleled in its ability to describe the qualitative characteristics of mouthfeel [33,34].

Gallic acid exhibited a potent, dose-dependent acidity, while showing no detectable bitterness or astringency (Figure 5D, Figure 6D and Figure 7D). Acidity is induced by protons (H^+^) released from food components. These protons activate the acid taste receptor, Otopetrin-1 (OTOP1), which is expressed in Type III taste receptor cells (TRCs). The activation of OTOP1 directly alters the membrane potential (*Vm*), leading to cell depolarization. This depolarization triggers the opening of voltage-gated Na^+^ channels, generating a series of action potentials that subsequently open voltage-gated Ca^2+^ channels. The resulting influx of calcium ions triggers the release of neurotransmitters—primarily serotonin—into the interneurons, ultimately eliciting the sensation of acidity [35]. The findings of this study suggest that protons derived from low-molecular-weight phenolic acids, such as gallic acid, contribute significantly to the acidity of foods rather than to their bitterness or astringency.

Quercetin exists in plants as either an aglycone or a glycoside; however, the aglycone form is poorly soluble in water. Quercetin hydrate does not exhibit acidity, bitterness, or astringency (Figure 5E, Figure 6E and Figure 7E) in the present study. This compound is generally considered a bitter compound [36] and is listed in the Bitter Database (https://bitterdb.agri.huji.ac.il/dbbitter.php, accessed on 10 April 2025). Furthermore, in vitro cell-based assays have reported its interactions with multiple bitter taste receptors, including T2R4, 7, 10, 14, 39, 40, 43, 44, and 46 [37]. While most quercetin in nature exists as glycosides, reports regarding their sensory properties remain scarce [38]. Notably, quercetin glycosides possess higher water solubility than the aglycone, which may significantly influence the expression of their taste profiles. The lack of perceived taste in this study is likely attributable to the low solubility of the quercetin aglycone, which prevented it from achieving the bioavailable concentration necessary to activate the aforementioned T2Rs under these specific aqueous conditions required to activate the aforementioned T2Rs.

It should be noted that the sensory profile of quercetin hydrate—which was characterized by a lack of distinct bitterness or astringency in this study—may be influenced by its poor aqueous solubility. At 0.4 mg/mL, quercetin likely existed as a suspension. Since the activation of taste receptors requires the dissolution of compounds in saliva, the limited solubility of quercetin may have prevented sufficient molecular interaction with T2R receptors. Therefore, our findings regarding quercetin should be interpreted as the sensory impact of its aqueous dispersion at this specific concentration, rather than its intrinsic taste profile in a fully solubilized state. Future studies using food-grade solubilizers or different delivery systems are necessary to further clarify the organoleptic properties of flavonols.

EGCG is present in high concentrations in green tea primarily as an aglycone. It exhibits high water solubility and is well-recognized for its potent biological activities. In the present study, EGCG exhibited strong, dose-dependent bitterness (Figure 6F), while a slight astringency was also perceived across all tested concentrations (Figure 7F).

In vitro studies have reported that EGCG interacts with various bitter taste receptors, including T2R4, 7, 10, 14, 39, 40, 43, 44, and 46 [37]. Furthermore, our previous computational chemistry analysis revealed a robust interaction between EGCG and T2R46, yielding a binding energy of −7.5815 kcal/mol. This interaction is notably stronger than that of the positive control, strychnine (−6.0767 kcal/mol) [39]. Since bitterness and astringency are defining characteristics of green tea, these findings further support the suggestion that gallate-type catechins, particularly EGCG, are primary contributors to these sensory profiles [40].

The PRF used in this study contained approximately 39% of identified procyanidin oligomers and 17.5% catechins. The remaining mass likely consists of higher-order procyanidin polymers that are not easily quantified by conventional HPLC. Although the presence of monomeric catechins in this fraction cannot be ignored, the distinctively high intensity of astringency observed for the PRF—which exceeded that of the pure EGCG sample—indicates the significant sensory contribution of the procyanidin oligomers and polymers. However, we acknowledge that synergistic effects between these components may exist, and the results should be interpreted as the collective sensory profile of a botanical polyphenol fraction rather than a specific procyanidin molecule.

Procyanidins are oligomers of catechins, with molecular weights ranging from 600 Da for dimers to over 6000 Da for polymers with 20 or more units. They are prevalent in various foods; notably, cocoa contains high concentrations of B-type procyanidins. In this study, the PRF exhibited bitterness across all tested concentrations (Figure 6G). Previous in vitro studies have reported that dimeric procyanidin B2 activates T2R5 and T2R39, while trimeric procyanidin C1 activates T2R5 [41]. Our previous molecular docking simulations analyzing the interactions between procyanidins and T2R46 revealed that procyanidins B2 and C1 also exhibited significantly stronger affinities, with binding energies of −8.7455 kcal/mol and −11.0433 kcal/mol, respectively, compared to the positive control strychnine (−6.0767 kcal/mol) [39]. Furthermore, the PRF exhibited a significant dose-dependent response in astringency (Figure 7G). As previously noted, astringency is a sensory attribute observed only in specific polyphenols, and the precise receptor-mediated or physiological mechanisms underlying its perception remain largely elusive. By applying the sensory evaluation methodology established in this study to characterize astringent polyphenols, it may be possible to clarify the cognitive and physiological processes of astringency in relation to their specific chemical properties.

Regarding the panel size, this study was conducted as a pilot study with a panel of seven trained individuals. While a sample size of seven to twelve is generally considered acceptable for descriptive analysis (DA) when the panelists are highly trained [1], we acknowledge that a smaller panel may limit the generalizability of the findings and increase the influence of individual sensory thresholds on the group mean. To mitigate these limitations, we implemented an intensive four-day training program to minimize inter-individual variability and ensure panel alignment, as evidenced by the significant reduction in taste recognition thresholds (TRTT) shown in Figure 2. Although the post hoc power analysis confirmed sufficient statistical power (1 − β > 0.8) to detect the large effect sizes observed in this study, future research involving a larger, more diverse consumer panel would be beneficial to validate these results across a broader population.

Furthermore, while measures were taken to mitigate sensory fatigue, such as intervals between sessions and mouth rinsing, the intensive nature of the four-day training and multiple daily assessments may have led to some degree of sensory adaptation. This remains a limitation of the current pilot study and may account for a portion of the variance observed in the scoring of complex polyphenol mixtures.

In this pilot study, we used a newly established rigorous sensory evaluation protocol to quantitatively demonstrate for the first time that there are clear differences in the sensory characteristics of representative polyphenols. These differences in sensory characteristics are directly attributable to specific chemical structures, such as the carboxyl groups of phenolic acids and the degree of polymerization of flavan-3-ols. These sensory characteristics play the most fundamental role in determining food preferences and value, making the approach of this study highly significant. Furthermore, as recent research has shown, the sensory characteristics of these compounds may be intricately linked to their diverse health-promoting functions when ingested [2,3,42].

However, a notable limitation of this study is the impracticality of evaluating the functional properties of all 490 polyphenols documented in the Phenol-Explorer database (http://phenol-explorer.eu/, accessed on 10 April 2025) using the current methodology. The primary cause of this constraint is the difficulty in procuring many of these compounds; even when available, they are often provided in quantities insufficient for sensory testing or exhibit poor solubility in experimental solvents.

In conjunction with conducting recurrent sensory evaluations on a more extensive array of available polyphenols by employing this research methodology, multivariate statistical analysis, such as cluster analysis, will be utilized to categorize polyphenols into distinct subfamilies, based on the data obtained. Furthermore, our ultimate goal is to construct quantitative structure–activity relationships, or machine learning will be employed to construct robust sensory prediction models, thereby enabling a systematic understanding of the sensory properties of polyphenols.

## 5. Conclusions

In this study, we established a robust training and evaluation framework for identifying the sensory attributes and intensities of acidity, bitterness, and astringency in polyphenols by integrating FPA, 3-AFC, and QDA. Using this methodology, we evaluated four types of polyphenols with distinct chemical structures, revealing that each possesses a unique sensory profile.

Within the scope of this pilot study, our results demonstrated that specific structural features—such as the phenolic acid structure in gallic acid and the flavan-3-ol skeleton in EGCG—were associated with distinct sensory profiles like acidity and bitterness, respectively. However, since these inferences are based on a limited set of four compounds, they should be interpreted with caution and not be generalized to all members of these polyphenol classes. Further investigation using a broader range of chemical structures is required to validate whether these findings represent universal structure–sensory relationships. These results suggest that the proposed method can quantitatively assess the sensory characteristics of polyphenols that influence food palatability. Future research will further elucidate the relationship between these sensory attributes and their specific chemical structures. Moreover, given that recent studies indicate interactions between polyphenols and taste or nociceptors may induce various physiological changes, this methodology holds potential as a predictive tool for the health-promoting activities associated with polyphenol intake. Therefore, the methodology and findings of this study provide a crucial foundation for developing next-generation functional foods that target health maintenance via sensory nutrition-mediated physiological pathways.

## Figures and Tables

**Table 1:** Concentrations of polyphenols in epigallocatechin (EGCG) or procyanidin rich fraction (PRF).

**Epigallocatechin Gallate**	**Concentration (mg/g)**
theobromine	n.d.
caffeine	<0.01
epigallocatechin gallate	948.0
**Procyanidin-Rich Fraction** **(PRF)**	**Concentration (mg/g)**
theobromine	3.8
caffeine	n.d.
(+)-catechin	11.7
(−)-epicatechin	164.2
procyanidin B2	117.5
procyanidin B5	20.1
procyanidin C1	50.3
cinnamtannin A2	25.9

The concentrations of all compounds were determined by HPLC method.

**Table 2:** Terms using flavor profiling analysis (FPA) test *.

Terms	Definition of Terms
sweetness	Taste sensation stimulated by sugars such as sucrose and other substances such as saccharin i.e., sucrose 5%
salty	Taste caused by substances such as table salt
acidity	Taste sensation stimulated by acids contained in citric fruits such as lemon, i.e., citric acid 0.15%
bitterness	Taste sensation associated with caffeine in a water solution, i.e., caffeine 0.15%
umami	Taste is caused by substances such as monosodium L-glutamate and sodium 5′-inosinate.
astringency	A combination of shrinking, puckering, drying, and roughening sensations in the mouth
dryness	A dry mouthfeel that lacks moisture.
roughness	Sensation of roughness in mouth
puckering	Sensation of contraction, puckering in mouth
numbness	Sensation of numbness in mouth
pungency	Sensation is caused in the mouth by substances such as capsaicin in chili peppers.
oily	Sensation of oily in mouth

* This table of terms was created according to the previous papers [25,26].

**Table 3:** Results of the sensory tests for the standards and the polyphenols at 0.4 mg/mL using profiling analysis (FPA) and quantitative descriptive analysis (QDA).

Citric acid (Standard for acidity)			Caffeine (Standard for bitterness)		
	before training	after training		before training	after training
	Mean ± SD	Mean ± SD		Mean ± SD	Mean ± SD
Sweetness	0.00 ± 0.00	0.00 ± 0.00	Sweetness	0.00 ± 0.00	0.00 ± 0.00
Saltiness	0.00 ± 0.00	0.00 ± 0.00	Saltiness	0.00 ± 0.00	0.00 ± 0.00
Acidity	50.29 ± 27.64	61.29 ± 12.85	Acidity	0.00 ± 0.00	0.00 ± 0.00
Bitterness	0.00 ± 0.00	0.00 ± 0.00	Bitterness	53.00 ± 22.74	15.71 ± 18.21 *
Umami	0.00 ± 0.00	0.00 ± 0.00	Umami	0.00 ± 0.00	0.00 ± 0.00
Astringency	11.14 ± 29.48	0.00 ± 0.00	Astringency	7.00 ± 18.52	0.43 ± 1.13
Dryness	0.00 ± 0.00	0.00 ± 0.00	Dryness	0.00 ± 0.00	0.00 ± 0.00
Roughness	0.00 ± 0.00	0.00 ± 0.00	Roughness	0.00 ± 0.00	0.00 ± 0.00
Puckering	0.00 ± 0.00	0.00 ± 0.00	Puckering	4.00 ± 10.58	0.00 ± 0.00
Numbness	0.00 ± 0.00	0.00 ± 0.00	Numbness	3.29 ± 8.69	0.00 ± 0.00
Pungency	0.00 ± 0.00	0.00 ± 0.00	Pungency	0.00 ± 0.00	0.00 ± 0.00
Oily	0.00 ± 0.00	0.00 ± 0.00	Oily	0.00 ± 0.00	0.00 ± 0.00
PAS, Potassium aluminum sulfate (Standard for astringency)					
	before training	after training			
	Mean ± SD	Mean ± SD			
Sweetness	0.00 ± 0.00	0.86 ± 2.27			
Saltiness	0.00 ± 0.00	0.00 ± 0.00			
Acidity	0.00 ± 0.00	6.43 ± 7.59			
Bitterness	0.00 ± 0.00	1.14 ± 3.02			
Umami	0.00 ± 0.00	0.00 ± 0.00			
Astringency	50.57 ± 26.37	23.86 ± 18.05			
Dryness	0.00 ± 0.00	0.00 ± 0.00			
Roughness	0.00 ± 0.00	0.00 ± 0.00			
Puckering	13.14 ± 28.00	1.29 ± 3.40			
Numbness	0.00 ± 0.00	0.00 ± 0.00			
Pungency	0.00 ± 0.00	0.00 ± 0.00			
Oily	0.00 ± 0.00	0.00 ± 0.00			
Quercetin hydrate			Gallic acid		
	before training	after training		before training	after training
	Mean ± SD	Mean ± SD		Mean ± SD	Mean ± SD
Sweetness	1.00 ± 2.65	0.00 ± 0.00	Sweetness	0.00 ± 0.00	0.29 ± 0.76
Saltiness	0.00 ± 0.00	0.00 ± 0.00	Saltiness	1.00 ± 2.65	0.00 ± 0.00
Acidity	0.00 ± 0.00	0.00 ± 0.00	Acidity	29.57 ± 19.03	18.71 ± 5.94
Bitterness	4.43 ± 4.28	3.14 ± 4.30	Bitterness	6.86 ± 18.14	0.43 ± 1.13
Umami	0.00 ± 0.00	0.00 ± 0.00	Umami	0.00 ± 0.00	0.00 ± 0.00
Astringency	0.14 ± 0.38	0.71 ± 1.89	Astringency	14.14 ± 14.69	3.29 ± 6.45
Dryness	0.00 ± 0.00	0.00 ± 0.00	Dryness	0.00 ± 0.00	0.00 ± 0.00
Roughness	0.00 ± 0.00	0.00 ± 0.00	Roughness	1.86 ± 4.91	0.00 ± 0.00
Puckering	0.00 ± 0.00	0.00 ± 0.00	Puckering	12.00 ± 12.30	3.00 ± 6.35
Numbness	0.00 ± 0.00	0.00 ± 0.00	Numbness	0.00 ± 0.00	0.00 ± 0.00
Pungency	0.00 ± 0.00	0.00 ± 0.00	Pungency	0.00 ± 0.00	0.00 ± 0.00
Oily	1.00 ± 2.65	0.43 ± 1.13	Oily	0.00 ± 0.00	0.00 ± 0.00
EGCG			Procyanidin rich fraction		
	before training	after training		before training	after training
	Mean ± SD	Mean ± SD		Mean ± SD	Mean ± SD
Sweetness	0.00 ± 0.00	0.00 ± 0.00	Sweetness	0.00 ± 0.00	0.00 ± 0.00
Saltiness	0.00 ± 0.00	0.00 ± 0.00	Saltiness	0.00 ± 0.00	0.00 ± 0.00
Acidity	0.00 ± 0.00	0.00 ± 0.00	Acidity	0.00 ± 0.00	0.00 ± 0.00
Bitterness	63.29 ± 21.62	39.00 ± 20.07 *	Bitterness	50.71 ± 33.44	30.86 ± 26.50
Umami	0.00 ± 0.00	0.00 ± 0.00	Umami	0.00 ± 0.00	0.00 ± 0.00
Astringency	0.86 ± 2.27	11.86 ± 22.88	Astringency	42.00 ± 30.34	20.86 ± 17.39
Dryness	0.00 ± 0.00	0.00 ± 0.00	Dryness	0.00 ± 0.00	0.00 ± 0.00
Roughness	1.86 ± 4.91	0.00 ± 0.00	Roughness	0.00 ± 0.00	0.00 ± 0.00
Puckering	6.71 ± 12.97	0.00 ± 0.00	Puckering	20.00 ± 30.49	3.14 ± 8.32
Numbness	0.00 ± 0.00	0.00 ± 0.00	Numbness	1.29 ± 3.40	0.00 ± 0.00
Pungency	0.00 ± 0.00	0.00 ± 0.00	Pungency	0.00 ± 0.00	0.00 ± 0.00
Oily	0.00 ± 0.00	0.00 ± 0.00	Oily	0.00 ± 0.00	0.00 ± 0.00

The data were shown as mean and standard deviation (each *n* = 7). All statistical analysis was performed using Kruskal-Wallis test, and Dunn’s multiple comparison test was used for post hoc comparisons. * *p* < 0.05.

## Data Availability

The original contributions presented in the study are included in the article. Further inquiries can be directed to the corresponding author.
